# Cyclin-Dependent Kinase Inhibition in Prostate Cancer: Past, Present, and Future

**DOI:** 10.3390/cancers17050774

**Published:** 2025-02-24

**Authors:** Matthew Siskin, Minas P. Economides, David R. Wise

**Affiliations:** Genitourinary Medical Oncology Service, Perlmutter Cancer Center, NYU Langone Heath Center, New York, NY 10016, USA; matthew.siskin@nyulangone.org (M.S.); minas.economides@nyulangone.org (M.P.E.)

**Keywords:** prostate cancer, cyclin-dependent kinase 4/6 inhibitors, cell-cycle inhibitors, targeted therapy

## Abstract

Background: Despite significant progress, prostate cancer remains a leading cause of death. Cyclin-dependent kinase (CDK) 4/6 inhibitors, which are already approved for the treatment of hormone receptor-positive breast cancer, are undergoing extensive testing as monotherapy and in various combinations as a potentially valuable treatment modality in prostate cancer patients. Thus far, a limited number of these studies have published results, which have been largely disappointing. Areas Covered: In this review, we describe the biologic rationale for the use of CDK4/6 inhibitors in prostate cancer, the existing clinical data describing their use in prostate cancer, and ongoing clinical trials of CDK4/6 inhibitors as monotherapy and in combination for the treatment of prostate cancer. In particular, we focus on possible resistance mechanisms that may be particularly relevant in prostate cancer patients, leading to de novo and acquired resistance, and we highlight novel strategies that can overcome this resistance. Conclusions: Current clinical trials are actively working to (1) refine the role of CDK4/6 inhibitors in prostate cancer patients; (2) develop new inhibitors of other cell-cycle targets, such as CDK2 and CDK7; and (3) explore novel combination therapies with inhibitors of other relevant pathways, such as PI3K or MAPK. Further genomic subtyping of advanced prostate cancer will likely shed light on the subsets of patients most likely to benefit from cell-cycle-targeted agents.

## Simple Summary

Despite significant progress, prostate cancer remains a leading cause of death. Cyclin-dependent kinase (CDK) 4/6 inhibitors, which are already approved for use in breast cancer, are undergoing extensive testing in prostate cancer patients. Thus far, a limited number of these studies have published results, which have been largely disappointing. This review summarizes the rational and existing clinical data on CDK4/6 inhibitors in prostate cancer. We explore possible reasons that current trials have failed to demonstrate significant benefits in prostate cancer patients and review possible strategies for overcoming treatment resistance.

## 1. Introduction

During the past decade, systemic treatment options for patients with advanced castration-resistant prostate cancer have expanded with the introduction and approval of the prostate-specific membrane antigen (PSMA)-targeted radiopharmaceutical lutetium Lu 177 vipivotide tetraxetan; the poly ADT ribose polymerase (PARP) inhibitors Olaparib, Rucaparib, Talazoparib, and Niraparib for homologous recombination-deficient (HRD) prostate cancer; and the checkpoint inhibitors nivolumab and pembrolizumab for mismatch repair-deficient (MSI-H) prostate cancer. All of these therapies have shown significant improvements in patient outcomes when added to androgen-deprivation therapy (ADT) in various clinical circumstances [1]. Despite these advances, prostate cancer remains the second leading cause of cancer-related mortality among men in the United States [2].

There is growing interest in identifying new molecular drivers that could be targetable in prostate cancer. The family of genes involved in cell cycle regulation is known to be the driver of the most aggressive advanced prostate cancers, and strategies targeting the cell cycle are an area of major unmet need [3,4]. Cyclin-dependent kinase (CDK) inhibitors, which are heavily utilized in breast cancer, are being actively explored to target the cell cycle in prostate cancer in several clinical trials. In breast cancer, estrogen receptor signaling upregulates the CDK4/6 pathway, leading to progression of the cell from the G1 to S phase and contributing to tumor growth. Abemaciclib, ribociclib, and palbociclib are all CDK4/6 inhibitors that are currently approved in the United States and have been shown to improve the clinical outcomes for patients with breast cancer when combined with endocrine therapy [5]. Whether a similar pathophysiology can be exploited in prostate cancer remains an unanswered question.

In this review, we will describe the mechanisms regulating cell cycle entry and transition, the available therapeutics that target these mechanisms, and the corresponding active clinical trials that are examining the safety and efficacy of these strategies in prostate cancer patients. We will also explore possible mechanisms of resistance to CDK4/6 inhibitors that may be particularly relevant in prostate cancer patients and the investigational agents that may be utilized to overcome these resistance pathways.

## 2. Cell Cycle Pathway in Normal Cells

Cellular division is a critical feature of normal human cells and is utilized both for growth and to replace damaged cells. In normal somatic tissues, the cell cycle is broadly divided into interphase and mitosis. Interphase is divided into the G1, S, and G2 phases. The G1 phase takes place during the gap of time between mitosis and DNA synthesis. In the G1 phase, RNA and protein synthesis lead to growth and replication of cellular structures. During this phase, some cells are capable of halting cell division and entering a quiescent phase called G0. Cells that proceed to division move from G1 to the S phase, and while in the S phase, DNA is replicated. Subsequently, the G2 phase takes place during the gap of time between DNA synthesis and mitosis. During this phase, the cell ensures that DNA synthesis and nuclear integrity are complete prior to proceeding with mitosis, at which time two identical daughter cells are produced [6].

**Figure 1:**
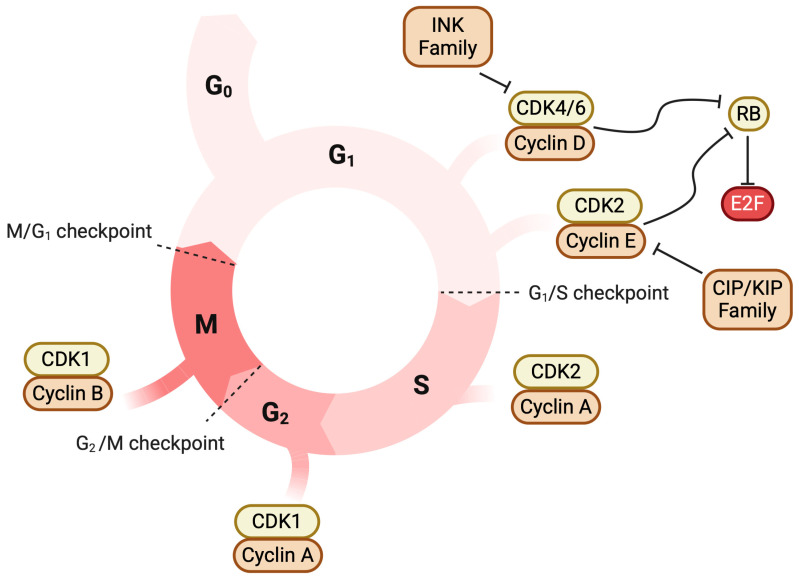
Overview of CDK–cyclin interaction in the cell cycle. Abbreviation: CDK, cyclin-dependent kinase. Normal cell cycle regulation signaling of prostate cells. CDK4/6–cyclin D1 and CDK2–cyclin E promote cell cycle progression from the G1 to S phase by inhibiting Rb-mediated inhibition of the transcription factor E2F. Other combinations of cyclin and CDK complexes mediate progression through other parts of the cell cycle. Some cells are capable of halting cell division and entering a quiescent phase called G0. The INK and CIP/KIP family proteins are negative regulators of CDK4/6 and CDK2 [8,9]. Created in BioRender. Siskin, M. (2025) https://BioRender.com/k71i916 accessed on 18 February 2025.

The cell cycle is highly regulated through a host of factors including cyclins, cyclin-dependent kinases, cyclin-dependent kinase inhibitors, and other regulatory elements [7]. A series of checkpoints exists between the major phases of the cell cycle that prevents the cell from dividing inappropriately. Cyclin levels rise and fall during different parts of the cell cycle and influence the activity of CDKs, which are present at stable levels throughout the cell cycle. Cyclin D and E rise during the G1 phase, while cyclin A rises during the S phase and G2 phase, and cyclin B rises during the M phase. Cyclin D production is regulated by the mitogen-activated protein kinase (MAPK) and phosphoinositide 3-kinase (PI3K) pathways. Various mechanisms that survey the integrity of DNA interface with this system and allow for further regulation of the cell cycle. For example, DNA damage can lead to the activation of the ataxia-telangiectasia mutated (ATM) and ataxia-telangiectasia and rad-3 related (ATR) kinases, which in turn activates checkpoint kinase 1 (CHK1) and checkpoint kinase 2 (CHK2), which can inhibit the cell cycle through multiple mechanisms. One particularly important mechanism is the phosphorylation of p53, which prevents p53 degradation and allows p53 to function as a transcription factor to hundreds of genes, resulting in the production of several cell-cycle inhibitors such as p21, which inhibits the cyclin D–CDK4/6 complex, which is critical to the G1/S checkpoint [8,9]. During the G1/S checkpoint, rising levels of cyclin D activate CDK4/6, while rising levels of cyclin E activate CDK2, forming active kinase complexes that phosphorylate the retinoblastoma protein (Rb), releasing Rb’s inhibition of the transcription factor E2F. In addition to the factors described above, this process is regulated by cell cycle division protein 25a (CDC25A), INK4 family proteins, and CIP/KIP family proteins. Once disinhibited, E2F can activate the transcription of genes needed to begin the S phase [6,8,10,11]. The cyclin A–CDK2 complex is needed to progress through the S phase, while the cyclin B–CDK1 complex is required to progress through mitosis [8,12,13]. A variety of other regulatory elements are involved in either inhibiting or promoting these processes and are critical to normal cell division; they have been thoroughly summarized elsewhere [6,8,14]. The major cell cycle checkpoints are summarized in Figure 1.

## 3. Dysregulation of the Cell Cycle Pathway in Cancer

Cell cycle dysregulation is an established hallmark of cancer. Cancer cells frequently mutate pathways that prevent cell cycle exit. Importantly, while some cell cycle checkpoints are commonly mutated in cancer cells, others are very rarely altered, implying that some degree of cell cycle regulation is critical even in cancer cells. Mutations in the p53 and E2F pathways, which are essential components of the G1/S checkpoint, are common across cancers [14,15,16].

Retinoblastoma was among the first described tumor suppressors. While it was first described in the disease that bears its name, dysregulation of this pathway has since been found to occur across multiple malignancies [17,18]. CDK4/6 amplification; cyclin D1 constitutive expression; CDC25 overexpression; *CDKN2A* (p16 INK4a) mutations; other INK4 and *CIP/KIP* mutations; and loss of function in *RB1*, *ATM*, *ATR*, *CHK1*, *CHK2*, and *TP53* mutations have all been described [8,19]. Interestingly, there are emerging data that demonstrate that different tumor subtypes have different levels of reliance on various components of the cell cycle, and this may partially explain variations in sensitivity to CDK inhibitors [20].

## 4. The Cell Cycle Pathway in Prostate Cancer

The mutational landscape of prostate cancer is complex, broad, and includes a wide variety of mutations in tumor suppressors and oncogenes [3,4,21,22,23,24,25,26]. A complete understanding of prostate cancer pathophysiology at the molecular level remains elusive, but over the last several years, the molecular pathways of prostate cancer have become increasingly understood and are being actively targeted across clinical trials [27]. Germline mutations are present in ~15% of patients with advanced prostate cancer and can involve homologous recombination repair (HRR) genes and mismatch repair (MMR) genes [21]. The most common somatic genomic alterations in prostate cancer include chromosome 8p loss (resulting in the loss of NKX3.1), 10q loss (resulting in the loss of PTEN), 17q loss (resulting in the loss of TP53), 8q gain (resulting in Myc overexpression), androgen receptor alterations, SPOP mutations, and TMPRSS2-ETS gene fusions such as TMPRSS2-ERG. Many of these mutations ultimately impact prostate cancer growth via either direct or indirect modulation of the cell cycle, and cell cycle mutations have been shown to be correlated with poor prognosis among patients with hormone-sensitive and hormone-resistant disease [3,4,22,23,24,25,26].

Homologous recombination repair (HRR) mutations are common in metastatic castration-resistant prostate cancer (mCRPC). Breast cancer gene 2 (BRCA2) is the most frequently impacted HRR component, and mutations in this gene occur in 10–13% of mCRPC cases. Mutations in other components of this pathway such as ATM, PALB2, and CHEK2 occur less frequently [28,29]. The molecular biology of HRR is complex and has been thoroughly summarized elsewhere. Ultimately, this pathway can impact cell cycle regulation via BRCA1-mediated phosphorylation of p53 by ATM/ATR, resulting in inhibition of the CDK4/6–cyclin D complex at the level of the G1/S checkpoint [30]. Furthermore, 5% of metastatic prostate cancer patients are mismatch repair deficient, with the majority of these cases involving somatic mutations in MMR genes rather than germline mutations. The MMR pathway is also capable of inducing cell cycle arrest via ATM/ATR signaling [31,32,33].

Other common somatic mutations in prostate cancer often impact cellular growth pathways. TP53 and RB1 mutations occur in 33% and 11% of patients with mCRPC, respectively, and directly influence cell cycle regulation, as detailed above [34]. PTEN mutations, which are present in 20–40% of prostate cancers, occur more commonly in patients with TMPRSS2-ERG fusions. PTEN is a negative regulator of the PI3K/AKT/MTOR pathway that is capable of inducing p27 expression and reducing CDK2 activity, preventing progression through the G1/S checkpoint [35,36]. Notably, the TMPRSS2-ERG fusion, which is present in approximately 50% of prostate cancers, leads to an AR-mediated upregulation of ERG signaling and is implicated in pathways that lead to metastasis rather than cell growth [37].

Androgen receptor (AR) activity is a key component in the pathophysiology of prostate cancer and is a regulator of the transcription of genes necessary for cell cycle progression from the G1 to S phase. Upon ligand binding, the androgen receptor activates, forms a homodimer, and travels to the nucleus, where it acts as a transcription factor on the androgen-responsive elements of the genome [38,39]. In addition to its role as a transcription factor, AR signaling also acts in the cytoplasm to modulate a variety of signal transduction pathways [40]. AR activity in prostate cancer cells has been shown to activate the MAPK pathway as well as Src and to increase levels of cyclin D1 via a PI3K/mTOR-mediated mechanism [41,42,43,44,45]. The absence of AR activity has been shown to lead to reduced levels of cyclin D3 and cyclin A, reduced CDK4 and CDK2 activity, the hypophosphorylation of Rb, and G1 arrest in prostate cancer cells [46]. In addition, AR activity may facilitate CDK2 activation through the degradation of p27, resulting in Rb phosphorylation and progression through the G1/S checkpoint [38,46]. Overall, these studies highlight the potential of AR activity to stimulate growth, in part, by directly activating the core components of cell cycle progression.

However, these findings have not been consistently shown across preclinical models. In other prostate cell lines, androgen activity has been shown to repress growth; reduce CDK4/6, CDK2, cyclin D1, and D2 transcription; increase the degradation of cyclin D1 mRNA; increase CDKN1A and cyclin E2, A2, and B1-3 transcription; and reduce Rb phosphorylation, raising the possibility of differing patterns of AR interactions with cell cycle regulators in different cell lines [47]. While well-established treatment paradigms in prostate cancer clearly establish the therapeutic approach of testosterone reduction and AR inhibition in prostate cancer, it has also been known for many years that supraphysiologic levels of testosterone can paradoxically inhibit prostate cancer growth, pointing to a possible tumor suppressor function of AR activity in certain circumstances. Studies have shown that in the presence of high levels of androgens, AR activity can enhance the Rb1 tumor-suppressor function and downregulate c-myc tumors, leading to suppressed growth in prostate cancer cell lines [48,49].

**Figure 2:**
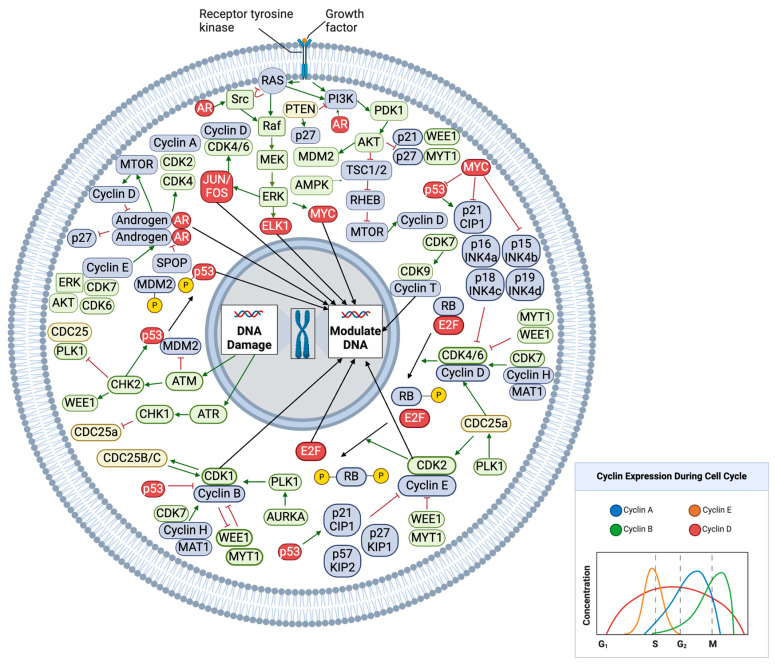
Overview of interactions between cell checkpoints, DNA damage responses, growth factor signaling pathways, and androgen receptor signaling.

Additionally, in contrast to the estrogen receptor (ER), which is activated by cyclin D1, the feedback interactions between the androgen receptor and the cyclin–CDK system are more complex [50,51]. Cyclin D1 inhibits androgen receptor activity through a variety of mechanisms [52,53,54,55,56]. This pattern of activity has led some authors to propose that a negative feedback loop exists whereby AR activity stimulates cyclin D expression to promote cell cycle progression but that this upregulation of cyclin D also attenuates further AR activity. Again, importantly, this feedback is unique to the AR, in contrast to the ER, which is activated by cyclin D1 [57] These interactions are further complicated by findings that indicate different isoforms of cyclin D have been shown to have differing effects on AR activity. While cyclin D1a represses AR activity, cyclin D1b appears to modulate AR activity in a distinct fashion that results in increased AR-dependent growth [58]. CDK6 has been shown to bind to and stimulate the transcriptional activity of the androgen receptor, particularly in androgen receptors bearing the T877A mutation, and CDK7-mediated activation of the mediator complex enhances androgen receptor transcription factor activity further, demonstrating how other components of the CDK–cyclin system also regulate AR activity [59,60]. Although androgen-deprivation therapy can influence these pathways, prostate cancer cells eventually become castration-resistant via various mechanisms that include AR mutations, AR amplification, and aberrant activation of AR [61]. SPOP mutations, which occur in approximately 10% of prostate cancers, seem to have an oncogenic role that is in part related to the impaired degradation of oncoproteins such as AR [62]. Figure 2 summarizes the interactions between the cell cycle checkpoints, DNA repair pathways, growth factor pathways such as MAPK and PI3K, and the AR signaling pathway.

In response to growth signaling from androgen receptor pathways as well as other growth factor pathways such as the MAPK and PI3K pathways, cyclin D levels rise at the beginning of the cell cycle and form a complex with CDK4 and CDK6. This complex is activated by the CDK-activating kinase (CAK), which is composed of CDK7, Cyclin H, and MAT1. PLK1 activates CDC25a, which is another activator of this complex, while the INK family proteins and WEE1/MYT1 inhibit cyclin D–CDK4/6 activity. Cyclin D–CDK4/6 phosphorylates Rb, leading to reduced affinity for E2F. The cyclin E–CDK2 complex further phosphorylates Rb, disinhibiting E2F, which subsequently travels to the nucleus to modulate transcription. The cyclin E–CDK2 complex is activated by CDC25a and inhibited by WEE1/MYT1 as well as the KIP/CIP family proteins. Later in the cell cycle, rising levels of cyclin B leads to complex formation with CDK1. This complex is activated by CAK, PLK1, and CDC25B/C, while it is inhibited by WEE1/MYT1. AURKA modulates the activity of PLK1. CDK1–cyclin B then travels to the nucleus, activating multiple components required for mitosis. DNA damage can lead to cell cycle arrest via activation of ATM/ATR and CHK1/2, which modulate multiple components of the cell cycle. The MAPK, PI3K, and AR pathways all intersect with these cell cycle components at multiple points. MAPK pathway activation ultimately leads to activation of cyclin D–CDK4/6 as well as inhibition of cell cycle inhibitors including p53, CIP/KIP, and INK family proteins. The PI3K pathway similarly modulates cyclin D levels as well as CIP/KIP family proteins, p53, and WEE1/MYT1 activity. AR activity is capable of directly modulating cell cycle activity while also increasing activity in both the MAPK and PI3K pathways [8,9,30,35,38,39,40,41,42,43,44,45,46,52,53,54,55,56,57,62,63,64,65,66]. Created in BioRender. Siskin, M. (2025) https://BioRender.com/x07a420 accessed on 15 January 2025. Color Code: In the above figure components coded red have transcription factor activity, pale yellow components have phosphatase activity and green components have kinase activity. All other components are coded in blue. 

## 5. CDK4/6 Inhibitors in Breast Cancer

Following the publication of several pivotal clinical trials, CDK4/6 inhibitors emerged as the preferred first-line therapy in combination with hormone therapy in hormone receptor-positive, HER2-negative metastatic breast cancer. These oral, highly selective agents function via a mechanism of action that involves binding to the ATP cleft of CDK4/6, leading to inactivation of the complex formed by cyclin D and CDK4/6, and thus, resulting in enhanced Rb activity and cell cycle arrest [67]. Currently approved CDK4/6 inhibitors include palbociclib, ribociclib, and abemaciclib. The pharmacologic properties of these agents have been thoroughly summarized elsewhere [68]. Palbociclib was the first CDK4/6 inhibitor to be FDA approved in 2015 for the treatment of postmenopausal women with locally advanced or metastatic hormone receptor-positive, HER2-negative breast cancer [69]. The approval was based on the PALOMA-2 study, which demonstrated a doubling of the median progression-free survival (PFS) in women receiving combination palbociclib plus endocrine therapy over endocrine therapy alone [70]. The Monaleesa and Monarch trials demonstrated similar results and led to the FDA approval of ribociclib and abemaciclib, respectively [71,72,73]. The combination of endocrine therapy and CDK inhibition consistently improved PFS and response rate over endocrine therapy alone, with more recent data showing improvements in overall survival [74]. In addition, abemaciclib has been approved as monotherapy in patients with metastatic hormone receptor-positive HER2-negative breast cancer that has progressed through hormone therapy and chemotherapy based on the results of the Monarch 1 single-arm phase 2 trial, which demonstrated an objective response rate (ORR) of 19.7%, median PFS of 6 months, and median overall survival of 17.7 months [75].

The use of these agents has also entered the adjuvant setting for patients with localized disease. The Natalee trial evaluated the use of 3 years of adjuvant ribociclib in combination with an aromatase inhibitor in patients with stage 2 or stage 3 hormone receptor-positive, HER2-negative breast cancer and found that the addition of ribociclib significantly improved disease-free survival (DFS) [76]. The MonarchE trial similarly evaluated the use of 2 years of adjuvant abemaciclib in addition to hormone therapy in patients with nodal involvement and other high-risk features and found a significant improvement in DFS [77]. Abemaciclib and ribociclib are now approved for use in the adjuvant setting.

In the above trials, these agents were well tolerated, with notable side effects including fatigue, acute kidney injury, and hematologic toxicity such as neutropenia. In addition, ribociclib has been associated with qtc prolongation and liver injury, while abemaciclib has been associated with gastrointestinal toxicity such as nausea and diarrhea. Retrospective analyses published after the approval of these agents have also raised concern for increased venous thromboembolism risk [78].

## 6. CDK4/6 Inhibitors in Prostate Cancer

While CDK4/6 inhibitors have shown clear benefits in breast cancer in a range of clinical circumstances as detailed above, their use in prostate cancer is not yet established. As of this writing, there are no FDA-approved indications for the use of CDK4/6 inhibitors in prostate cancer patients. Whether the AR-mediated cellular growth seen in prostate cancer cells as well as other dysregulated cellular functions such as PI3K/AKT pathway activity and DNA repair dysfunction render prostate cancers susceptible to CDK4/6 inhibition is an open question. In the next sections, we will highlight recently published and active clinical trials exploring the use of these agents across the spectrum of prostate cancer. The active and published trials discussed are summarized in Table 1 and Table 2, respectively.

### 6.1. CDK4/6 Inhibitors in Localized Prostate Cancer

CDK4/6 inhibitors are being actively explored in trials in high-risk localized patients in combination with radiation and surgical approaches. The potential benefit of exploring the efficacy of these agents in the neoadjuvant setting is that if biological activity is demonstrated in the surgical specimens obtained at the time of prostatectomy, that activity can be used to justify the additional exploration of these agents in other settings. The rationale for combining CDK4/6 inhibitors with radiation is in part based on data supporting the importance of cyclin D1 in radio-sensitizing prostate cancers and the possibility that CDK4/6 inhibition may potentiate the effects of radiation [83,84].

Abemaciclib is being investigated in combination with radiation and ADT in patients with high-risk localized or locally advanced prostate cancer (NCT04298983). In this phase II clinical trial, abemaciclib will be initiated along with ADT 3 months prior to radiation initiation. Treatment will continue for 2 years. Abemaciclib is also being investigated in combination with darolutamide as a neoadjuvant therapy prior to prostatectomy in patients with high-risk localized prostate cancer (NCT05617885) with an intended primary outcome of pathological response rate. It is unclear if the trials involving abemaciclib will proceed, given recent reports of Lilly deciding to stop the development of abemaciclib in prostate cancer (discussed below).

Ribociclib is also being evaluated in the high-risk localized setting. In a randomized phase II trial from Australia and New Zealand, patients with high-risk localized prostate cancer who are slated to undergo prostatectomy will receive either neoadjuvant ribociclib daily for 21 days or placebo (ACTRN12618000354280). The primary outcome will be the frequency of a 50% reduction in the ki-67 proliferation index from pretreatment prostate biopsy compared with that present in the prostate cancer tissue obtained at the time of radical prostatectomy.

### 6.2. CDK4/6 Inhibitors in Metastatic Castration-Sensitive Prostate Cancer (mCSPC)

For patients with mCSPC, palbociclib has been studied in combination with ADT in a randomized phase II trial of patients with mCSPC having retained RB1 expression. The hypothesis of this study was that palbociclib would improve clinical outcomes, given the preclinical data supporting the use of CDK4/6 inhibitors in prostate cancer detailed above and the anticipated parallels between prostate cancer and breast cancer where clinical benefit has been clearly established. The addition of palbociclib did not impact the outcomes of these patients. In comparison with ADT monotherapy, patients treated with ADT and palbociclib had an identical rate of achieving a PSA under 4 at 28 weeks (80% vs. 80%; *p* = 0.87). The rate of patients achieving an undetectable PSA (50% vs. 43%; *p* = 0.5), a radiographic response (89% vs. 89%; *p* = 078), and 12-month biochemical PFS (69% vs. 74%; *p* = 0.72) did not significantly differ between the treatment groups. The authors theorized that a greater than anticipated PSA response in the control group may have masked subtle improved outcomes in the palbociclib arm. They also noted that CHAARTED was published shortly after the study began and that patients with more extensive disease who were candidates for chemotherapy were encouraged to receive docetaxel based on these results, which may have limited their participation in this trial. Of note, in the CHAARTED trial, the proportion of patients who achieved a PSA under 0.2 at 12 months was 16.8% in patients receiving ADT monotherapy. While this study did examine molecular features, the study was underpowered to determine if certain molecular features correlated with sensitivity to CDK4/6 inhibition [79,85].

Abemaciclib in combination with a novel hormonal agent was also under investigation in this setting. In the phase III Cyclone 3 trial, patients with high-volume mCSPC were to be randomized to receive androgen-deprivation therapy with abiraterone plus abemaciclib or placebo (NCT05288166). The primary outcome of this study was investigator-assessed rPFS, while the secondary outcomes were CRPC-free survival, OS, and time to pain progression. However, in the first quarter of 2024, Lilly announced that they would be ending the Cyclone 3 trial due to futility in an interim analysis and terminating the abemaciclib in prostate cancer program [86]. No results have yet been made available from the study.

Based on these two studies, the addition of CDK4/6 inhibitors to hormone therapy in the treatment of metastatic hormone-sensitive prostate cancer does not appear to clearly improve outcomes. This lack of efficacy may be due to intrinsic resistance of prostate cancer cells to CDK4/6 inhibition, which may occur via a variety of mechanisms, which we will explore in the coming sections. When results of the Cyclone 3 trial are ultimately released, it will be important to assess if Rb loss or other molecular features were associated with poor responses to assess if any subset of patients benefited from the addition of these agents. To our knowledge, no active clinical trial is currently exploring the use of CDK4/6 inhibitors in patients with mCSPC. Investigations into the use of these agents in patients with castration-resistant disease are ongoing and will be described in the following section.

### 6.3. CDK4/6 Inhibitors in Metastatic Castration-Resistant Prostate Cancer (mCRPC)

Most of the active clinical trials evaluating CDK4/6 inhibitors are in the mCRPC setting. They include CDK4/6 inhibitor monotherapy and combination therapy with different treatments.

#### 6.3.1. CDK4/6 Inhibitor Monotherapy

The Cyclone 1 trial was a phase II clinical trial evaluating abemaciclib monotherapy in patients with mCRPC who had progressed after a novel hormonal agent and two taxane-based chemotherapy regimens. The primary outcome was investigator-assessed ORR. The ORR was 6.8% with a 45.5% reported disease control rate and a median OS of 8.4 months [80]. The Monarch 1 trial, which was a similar trial evaluating single-agent abemaciclib in breast cancer patients, described above, reported an objective response rate (ORR) of 19.7%, median PFS of 6 months, and median overall survival of 17.7 months [75]. While there are significant limitations in cross-trial comparisons, especially across diseases, this finding again suggests a lower efficacy of CDK4/6 inhibitors in prostate cancer patients compared with breast cancer patients. Fundamental differences in the interactions between CDK4/6 and AR versus CDK4/6 with ER may account for these findings. Other possible mechanisms underlying prostate cancer resistance to CDK4/6 inhibition that may underpin these results will be explored in detail below and include upregulation of other growth factor pathways such as MAPK and PI3K, CDK2 overexpression, and loss of tumor suppressors such as Rb.

Palbociclib monotherapy is also being investigated in a phase II trial in a similar patient population (NCT02905318). This study is a phase 2 single-arm study of patients with mCRPC who have progressed through first-line androgen deprivation and novel hormonal androgen receptor signaling inhibitor (ARSI) therapy who will be treated with palbociclib monotherapy. The primary endpoint will be the clinical benefit rate, defined as either a decline in PSA of 50% or more, a radiographic complete response or partial response, or stable disease for 12 weeks or greater. Palbociclib is also being assessed as part of a multi-arm phase 2 basket study involving multiple tumor subtypes, including patients with mCRPC who would be eligible for palbociclib if they have CCND1, -2, -3, or CDK4/6 amplification with retained Rb expression by IHC with a planned primary outcome of ORR (NCT02465060).

#### 6.3.2. CDK4/6 Inhibitor Combination Therapy

CDK4/6 inhibitors are being explored in mCRPC in combination with several types of therapies. Trials assessing combinations with chemotherapy are based on the evidence of the synergistic activity of cytotoxic chemotherapy with CDK inhibitors in preclinical models [87]. In a recently published single-arm phase Ib/II trial evaluating the efficacy of ribociclib in combination with docetaxel in patients with metastatic castration-resistant prostate cancer (mCRPC) who progressed through first-line hormonal therapy, including an ARSI, the 6-month radiographic PFS with the combination was 65.8% in patients with mCRPC. The median radiographic PFS was 8.1 months [81]. The authors of this study point out that there are limited data on the clinical efficacy of docetaxel following ARSI therapy with which to frame these results. They compared their results with a small retrospective series, which noted a PFS of docetaxel ranging from 4.1 to 5.1 months, and concluded that ribociclib may improve the efficacy of docetaxel. However, in the PRESIDE study, patients who progressed through enzalutamide and ADT who went on to receive single-agent docetaxel had a median radiographic PFS of 8.3 months, which is similar to the efficacy seen in this combination trial [88]. Ultimately, randomized trials will be needed to convincingly argue that combination therapy improves outcomes and justifies the additional toxicity.

Other trials are assessing the combination of CDK4/6 inhibitors with ARSIs. The Cyclone 2 trial was a phase II/III randomized clinical trial with patients with mCRPC who had progressed on ADT. Patients were randomized to receive abiraterone plus abemaciclib versus placebo. Results were presented at ASCO in 2024. Radiographic median PFS, overall survival, and time to symptomatic progression did not differ significantly between the groups, and the study agents were discontinued more often in the combination arm due to adverse events. Molecular subset analysis has not been reported for this study thus far. Attention to the differential efficacy in patients with loss-of-function mutations in Rb1, amplifications in cell cycle components, or high Ki67% will be of interest to understand if and how to move forward with this agent in mCRPC [82]. Abemaciclib plus darolutamide is being assessed in NCT05999968, which is a phase 1b that will evaluate the safety of this combination in patients with mCRPC. NCT05617885, mentioned above, in addition to evaluating the use of abemaciclib with darolutamide as a neoadjuvant therapy in high-risk disease, also intends to enroll a phase 1 cohort of patients with CRPC with a primary outcome of maximum tolerated dose, dose-limiting toxicity, and recommended phase 2 dose. As above, it is unclear if this study will continue, given Lilly’s plans to stop all investigation of abemaciclib in prostate cancer, as detailed above [86]. Ribociclib is also being studied in combination with enzalutamide in a phase I/II clinical trial (NCT02555189). The patient population in this study includes patients with mCRPC who are chemotherapy and ARSI naïve. The primary outcome is dose-limiting toxicity and the proportion of patients with a PSA response greater than or equal to 50%. While the results will be interpretable by comparing them with the PREVAIL study, which assessed single-agent enzalutamide in a similar patient population, this patient population is increasingly not applicable in real-world settings, given that the majority of patients now receive ARSI therapy in the mCSPC setting [89,90]. If clinical activity is demonstrated, it will need to be verified in larger phase 3 studies, which should assess a population of patients who progressed through ARSI therapy.

Novel CDK4/6 inhibitors are also being assessed in combination with hormonal therapies. TQB3616, a CDK4/6 inhibitor with superior in vitro inhibitory activity relative to palbociclib and abemaciclib against CDK4/6 kinase [91], is being evaluated in a phase Ib/II clinical trial in combination with abiraterone and prednisone (NCT05156450). The patient population includes patients with mCRPC who have progressed on ADT monotherapy. Primary outcome is 12-month rPFS. Dalpiciclib is also a novel CDK4/6 inhibitor with similar pharmacologic properties to ribociclib, abemaciclib, and palbociclib and is approved in China; it is now being assessed in combination with abiraterone in patients with mCRPC (NCT06500247) in a single-arm phase 2 study with a primary outcome of the rate of PSA reduction by 50% or greater at 12 weeks. Lastly, PF-07220060, a selective CDK4 inhibitor that spares CDK6, is being assessed in combination with enzalutamide in patients with prostate cancer in a phase 1/2a study whose primary outcome is safety (NCT04557449). In preclinical models, this agent has had similar clinical efficacy to existing CDK4/6 inhibitors with less treatment-associated neutropenia [92].

Outside of chemotherapy and endocrine therapy-based combinations, CDK4/6 inhibitors are also being evaluated in combination with immune checkpoint inhibitors, radiopharmaceuticals, and PARPi. CDK4/6 inhibitors may increase tumor antigen presentation, leading to increased effector T-cell activation and reduced regulatory T-cell activity [93,94,95]. Of note, prolonged CDK4/6 inhibition has been noted to be immunosuppressive in animal models, prompting some authors to propose that properly timed short-term CDK4/6 treatment could potentiate immune checkpoint therapy [94]. Partially based on this evidence, abemaciclib is being studied in combination with atezolizumab in a phase II trial of patients with mCRPC (NCT04751929). One potential limitation of this study is the potentially immunosuppressive continuous-dosing scheme used in this study rather than the pulsed dosing schedule. The population will include patients who have progressed after at least one novel antiandrogen therapy and are not candidates for taxane-based chemotherapy. Primary outcome measures of this trial will include 6-month PFS, ORR, rate of dose-limiting toxicity (DLT), and rate of adverse events. The Uplift trial (NCT05113537) will evaluate abemaciclib in combination with Lu-PSMA-617, a radiopharmaceutical that was recently approved for the treatment of patients with mCRPC who have progressed on novel hormonal agents and chemotherapy. In this phase I/II study, abemaciclib will be given prior to Lu-PSMA-617 to evaluate if the CDK4/6 is able to upregulate prostate-specific membrane antigen (PSMA) expression in the cancer cells and, as such, potentiate the effects of Lu-PSMA-617. Patients who have progressed after at least one novel hormonal agent and one line of taxane-based chemotherapy will be included. Primary outcomes will include the recommended phase II dose (on the phase I part), the proportion of patients with DLTs, and the change in maximum standardized update value across three lesions on gallium Ga 68 gozetotide scan. Lastly, a phase 1b study evaluating patients with multiple tumor types, including mCRPC, will assess the combination of Talazoparib with palbociclib in patients with defects in HRR genes such as BRCA1/2 with a primary outcome of dose-limiting toxicities and adverse event rate (NCT04693468).

While many of the above trials are ongoing, it is important to note that as of this writing, the available CDK4/6 inhibitors have not demonstrated comparable efficacy in prostate cancer compared with their activity in breast cancer. The limited efficacy observed for ribociclib and abemaciclib argues for a broader mechanistic basis for this lack of activity in prostate cancer. In the following section, we will review the known mechanisms of resistance to CDK4/6 inhibition that may have particular relevance to prostate cancer patients and propose approaches to combating those mechanisms.

## 7. Resistance Mechanisms to CDK4/6 Inhibitors

While there is improvement in PFS and OS with the combination of CDK4/6 inhibitors with endocrine therapy in breast cancer patients, all the patients ultimately progress. The different mechanisms of resistance are an active area of research and have led to the development of new therapeutics. Resistance can generally occur through cyclin E1–CDK2 amplification, activation of other CDK pathways such as CDK7, loss of tumor suppressors such as Rb or PTEN, or activation of other growth pathways such as the PI3K or MAPK pathway. The active and published trials discussed below that involve prostate cancer patients are summarized in Table 3 and Table 4, respectively.

### 7.1. CDK2 Overexpression and the Development of CDK2 Inhibitors

As described above, cyclin E activates CDK2 and drives cell cycle progression independent of CDK4 and -6 [102]. Hyperactivation of the CDK2–cyclin E complex is one of the main mechanisms of resistance to CDK4/6 inhibitors and can occur via a variety of mechanisms [103]. Preclinical data support the role of high CCNE1 levels in the development of palbociclib resistance, with evidence of re-sensitization to CDK4/6 inhibition by genetic knockdown of CCNE1. When CDK4/6 is inhibited in breast cancer cells with low CCNE1, early adaptation occurs (up to 72 h), resulting in CDK2 activation and increased cyclin E2 (CCNE2) expression, eventually leading to S phase entry [104]. Next-generation sequencing of samples from patients who progressed after exposure to CDK4/6 inhibitors showed amplification of CCNE2 in 6 of 41 cases [105]. These patterns have been shown to clinically impact patient responses to CDK4/6 inhibition. From the transcriptional profiling of the PALOMA 3 trial, it was shown that samples expressing high CCNE1 were less responsive to palbociclib–fulvestrant combination therapy. The median PFS for patients with high versus low CCNE1 levels was 7.6 months vs. 14.1 months in the palbociclib [106]. All these data support the predisposition of CDK4/6-inhibited cells to activate CDK2. This mechanism may be particularly relevant in prostate cancer, given the reduced levels of p27, an inhibitor of CDK2, and increased levels of CDK2 and cyclin E have been known to be indicators of poor prognosis and associated with androgen independence in prostate cancer for several decades [107,108]. Olomoucine and NU2058, which are selective CDK2 inhibitors, were shown to be synergistic with antiandrogens in prostate cancer cells, providing further evidence for this as a possible therapeutic approach [109,110].

This acquired resistance mechanism has renewed interest in the development of CDK2 inhibitors. Cyclin E and CDK2 contribute to the phosphorylation of RB, thereby contributing to transition past the G1 S checkpoint. CDK2 is also involved in S phase progression via an interaction with cyclin A. CDK2 inhibition often produces S and G2 arrest and apoptosis [111]. CDK2 inhibition might also exert cytotoxic effects in CDK4/6 inhibitor-resistant cells that are driven by Rb loss [112]. One notable potential limitation to the efficacy of CDK2 inhibition stems from the ability of cyclin E to promote proliferation via CDK2-independent mechanisms and CDK1 compensating for CDK2 activity [113,114,115]. Despite this, CDK2 inhibitors are actively being investigated in clinical trials.

A combined CDK2/4/6 inhibitor is currently being tested in early-phase clinical trials [116]. PF-06873600 is being investigated as monotherapy and combined with endocrine therapy in patients with metastatic hormone receptor-positive, HER2-negative breast cancer, triple-negative breast cancer, or advanced ovarian cancer (NCT03519178). PF-07104091 is a novel selective CDK2 inhibitor that has been evaluated in a phase 1 study involving breast, gynecologic, and lung cancer patients [117]. Fadraciclib is a combined CDK2 and CDK9 inhibitor and is currently being investigated in a phase I/II clinical trial of patients with advanced solid tumors or lymphoma. CDK9 is a promising therapeutic target as well, as it regulates the transcription of certain genes, including cyclins, through the phosphorylation of RNA polymerase II [63]. The CYC065-101 study did not include a specific prostate cancer cohort, but it did include a tumor-agnostic basket cohort of patients with Myc or CCNE amplification. Phase 1 results were presented at ASCO in 2024, demonstrating safety, with further exploration ongoing (NCT04983810) [118]. BLU-222, another promising CDK2, has been evaluated in early-phase clinical trials in breast, gastric, and gynecologic malignancies, which demonstrated safety. Efficacy is continuing to be evaluated in ongoing clinical trials [119,120].

In prostate cancer, although there were promising preclinical data on the pan-CDK inhibitor flavopiridol, a phase II clinical trial in patients with advanced prostate cancer showed poor response rates when this agent was used as monotherapy and significant toxicity. The authors attributed the lack of efficacy to either an inappropriate dosing schedule or a simple lack of efficacy of the agent itself, given that multiple trials across multiple disease spaces, including patients with gastric cancer, also failed [96,121,122]. NUV-422 was a combined CDK2/4/6 inhibitor that was being evaluated in a phase Ib/II study in combination with enzalutamide in patients with mCRPC (NCT05191017). Nuvation bio discontinued development of this agent in August 2022 following the FDA placing several trials on clinical hold after patients developed uveitis that was not completely understood [123]. To our knowledge, no selective CDK2 inhibitor is being investigated in a prostate cancer-specific cohort currently.

### 7.2. CDK7 and the Development of CDK7 Inhibitors

CDK7 complexes with cyclin H and MAT1 to form the CDK-activating kinase (CAK), which activates multiple CDKs, including CDK4 and -6. CDK7 also complexes with other proteins and forms the TFIIH complex, which activates RNA polymerase and promotes transcription, which subsequently relies on the contribution of other CDKs such as CDK9, as mentioned above. the activation of CDK7 and CDK9 through Myc-driven pathways is another important mechanism of CDK4/6 inhibitor resistance that is beginning to be explored [63,64,65]. There are reasons to think that targeting this pathway may be particularly well suited to prostate cancer patients, given that CDK7-mediated activation of the mediator complex enhances androgen receptor transcription factor activity [60].

CDK7 inhibitors such as SY-1365 have begun to be explored in preclinical models, where they have shown promising anticancer properties [124]. Particularly intriguing are preclinical prostate cancer models where CDK7 inhibition with the newly identified inhibitor THZ1 attenuates AR signaling and eradicates hormone-sensitive and enzalutamide refractory prostate cancer cells [60,125,126].

Several CDK7 inhibitors are currently in clinical development. LY3405105, produced by Eli Lilly, was investigated in a phase I trial in patients with a range of advanced solid tumors. Notably, no prostate cancer cohort was included in their trial. While safety was adequate, limited clinical activity was noted, and the company aborted plans to further develop the agent [127]. In the QUARTZ trial, XL102, a CDK7 inhibitor produced by Exelixis, was planned to be evaluated as monotherapy and in combination with abiraterone in patients with mCRPC. However, 20% of patients experienced grade 3 treatment-related adverse events. Unfortunately, the company elected not to proceed with development of this agent, and further study has been terminated for business reasons [97,128]. Syros, which was previously developing SY-1365, mentioned above, stopped the development of this IV agent in favor of an oral CDK7 inhibitor with a superior pharmaceutical and safety profile [129]. The CDK7 inhibitor SY-5609, being developed by Syros pharmaceuticals, was also investigated in a phase I trial. The pancreatic cancer cohort data were recently presented and demonstrated adequate safety [130]. Samuraciclib, an oral selective CDK7 inhibitor being developed by Carrick Therapeutics, is being studied as monotherapy in patients with advanced solid tumors, including those with CRPC. Adequate safety has been demonstrated, with the most common adverse events being GI toxicity. Treatment response was demonstrated in a breast cancer cohort, and additional trials are ongoing in breast cancer patients, such as NCT05963984 and NCT05963997 [98]. Q901, which has been studied in patients with breast, gynecologic, lung, and pancreatic cancer, has also reported adequate tolerability in a phase 1 study [131].

CDK9 inhibitors have also shown preclinical activity in prostate cancer models [132]. In addition to fadraciclib, which was described above, other agents are being explored in early-phase clinical trials in other disease spaces, including hematologic malignancies and melanoma (NCT04588922, NCT03263637, NCT02745743, NCT00835419). One of the studies that has presented results is PRT2527, which was evaluated in multiple cancer types, including CRPC, and demonstrated reasonable tolerability, with efficacy data not yet reported [99].

### 7.3. Loss of Tumor Suppressor Genes

The PI3K/AKT pathway contributes to cell cycle progression via signaling that ultimately results in increased levels of cyclin D1 [133]. PTEN is a negative regulator of PI3K signaling, and PTEN loss has been shown to be a common mechanism of resistance to CDK4/6 inhibitors. PTEN loss leads to impaired p27 nuclear localization, leading to increased CDK2 and CDK4 activation [134,135].

In prostate cancer, alterations in this pathway have been shown to be relatively common among patients with mCRPC and to portend worse outcomes. PTEN loss allows PI3K/AKT activation to promote cell growth without AR signaling [136,137]. PTEN loss is present in approximately 40% of patients with mCRPC [23]. Real-world retrospective cohort studies have shown that PTEN loss of function in prostate cancer patients results in a 61% increased risk of death relative to patients with intact PTEN function [138]. Patients with these alterations may be predisposed to intrinsic resistance to CDK4/6 inhibition for reasons described above.

In fact, the combination of PI3K inhibitors with CDK4/6 inhibitors has led to tumor regression in breast cancer cell lines, leading to the investigation of this combination in clinical trials [104]. In October 2024, the FDA approved the combination of inavolisib, a PI3K inhibitor, with palbociclib and fulvestrant, based on the results of the Inavo120 study. In this phase 3 double-blind, randomized controlled trial, patients with PIK3CA mutated hormone receptor-positive, HER2-negative breast cancers who relapsed either during or within 1 year of completing adjuvant endocrine therapy were randomized to receive either inavolisib or placebo in combination with palbociclib and fulvestrant. The combination arm had a median PFS of 15 months vs. 7.3 months in the placebo group (HR 0.43; *p* < 0.001) [139]. These data clearly demonstrate the potential utility of combining PI3K inhibitors with CDK4/6 inhibitors. To our knowledge, this combination is not yet being assessed in prostate cancer patients. Of note, ongoing clinical trials are testing PI3K/AKT pathway inhibitors in combination with AR pathway inhibitors. The AKT inhibitor ipatasertib, when combined with abiraterone, showed promising activity with improved radiographic PFS in a randomized phase II trial of patients with mCRPC and PTEN loss [100]. Samotolisib, an inhibitor of PI3K and mTOR, has been assessed in a phase 1b/2 study in combination with enzalutamide in patients with mCRPC. Radiographic PFS was significantly longer with the combination compared with single-agent enzalutamide (10.2 vs. 5.5 months) [101]. A variety of PI3K/AKT inhibitors are currently under investigation in prostate cancer in combination with other therapies such as ARSIs, docetaxel, bicalutamide, and hydroxychloroquine (NCT06190899, NCT03218826, NCT05348577, NCT01480154, NCT04586270, NCT01251861). Whether CDK4/6 inhibition further improves outcomes in patients treated with combined AR and PI3K/AKT blockade is deserving of further exploration.

Acquired Rb loss is another known mechanism of resistance to CDK4/6 inhibition [135]. In the PALOMA 3 trial, 4.7% of patients developed on-treatment RB1 mutations, indicating that CDK4/6 inhibitor therapy may provide selective pressure, leading to the development of Rb loss in a subset of breast cancer patients [140]. RB1 loss is known to occur in approximately 5% of patients with mCSPC and 20% of patients with mCRPC and is correlated with worse survival outcomes [3,23,141]. Importantly, RB1 loss is also present in 70–90% of small-cell neuroendocrine carcinomas of the prostate, a particularly aggressive form of prostate cancer. Studies evaluating CDK4/6 inhibitors in prostate cancer should assess if selective pressure leads to increased RB1 loss and increased small-cell transformation in prostate cancer patients, as this would constitute a significant treatment-related adverse event [142,143].

Prostate cancer patients with RB1 loss may be predisposed to CDK4/6 inhibitor resistance. Strategies for overcoming intrinsic resistance to CDK4/6 inhibition due to RB loss include using these agents in the castration-sensitive setting, where these mutations are rare, or in combination with other agents. Aurora kinases are enzymes involved in the regulation of several steps of mitosis [144]. Rb loss has been shown to have synthetic lethality with aurora kinase deficiency, and cancer cells developing RB loss after CDK4/6 inhibition were recently found to be sensitive to a selective aurora kinase A inhibitor [145]. A study evaluating the efficacy of a multiagent kinase inhibitor called tinengotinib, which inhibits aurora kinase A, in combination with either abiraterone or enzalutamide in patients with mCRPC is ongoing (NCT06457919).

### 7.4. MAPK Pathway Reliance

The mitogen-activated protein kinase (MAPK) pathway is an important signaling pathway involved in regulating cell proliferation [66]. MAPK activation is a known mechanism of CDK4/6 inhibition [146]. In patients with hormone receptor-positive breast cancer, RAS-pathway activating mutations, including KRAS G12D, KRAS Q61L, HRAS K117N, and NRAS were found in 10% of cases who progressed on CDK4/6 inhibition [105]. In prostate cancer, amplifications of members of the MAPK pathway are present in more than 30% of mCRPC [147]. Resistant cancer cells have increased MAPK activation, leading to CDK4/6-RB bypass and disease progression. MAPK activation in this setting, however, confers sensitivity to MEK inhibitors [146]. Multiple clinical trials are evaluating the combination of CDK4/6 inhibitors with MEK inhibitors across different solid tumors, including patients with KRAS-mutated non-small-cell lung cancer (NCT03170206). In prostate cancer, trametinib is being evaluated as monotherapy (NCT02881242). There are no active clinical trials of CDK4/6 inhibitors with MEK inhibitors in prostate cancer.

## 8. Future Directions and Expert Opinion

CDK inhibitors are highly effective, targeted agents against the cell cycle machinery. Despite their significant activity in breast cancer, their efficacy in prostate cancer has been limited [70,71,72,73,74,75,79,80,81,82]. The key biologic differences accounting for this difference in efficacy between breast and prostate cancer remain unclear. Here, we highlight several hypotheses, including the high prevalence of alterations in the PI3K pathway, RB1 loss, and the distinct ways in which the AR interacts with the cell cycle machinery relative to the ER [3,23,50,51,52,53,54,55,56,57,58,59,60,141]. We also highlight alternative cell cycle targets, such as CDK2 and CDK7, as well as novel combination trials that remain under investigation, in which CDK4/6 is used to modulate the biology of the prostate cancer cell rather than effect a cytostatic change. Ultimately, based on the above data, we believe that novel combinations of cell cycle targeting agents, when applied to the correct subsets of prostate cancer patients, could be an effective therapeutic strategy.

In order to further develop these agents, we expect that further genomic subtyping of advanced prostate cancer will be needed in order to shed light on which subsets of patients are most likely to benefit from these therapies. In addition, we encourage the investigators of the negative trials we describe above to explore the mechanisms of resistance in their trials and search for molecularly defined subgroups of patients that may have benefited from cell cycle inhibition, as this work could be invaluable in guiding future clinical research. We anticipate that a better understanding of the mechanisms of intrinsic and acquired resistance to CDK4/6 inhibitors will lead to the development of improved therapeutic strategies that will ultimately benefit patients with prostate cancer, either by refining the role of the CDK4/6 inhibitors in this patient population or by informing the development of new cell cycle inhibitors and novel combination therapies with inhibitors of other interconnected pathways.

## Figures and Tables

**Table 1:** Relevant active clinical trials of CDK4/6 inhibitors in advanced prostate cancer.

Agent (Trial Start Date)	Clinical Phase	Description of Trial	ClinicalTrials.gov Identifier	Primary Outcome
Ribociclib (2018)	II	Ribociclib prior to prostatectomy in high-risk prostate cancer	ACTRN12618000354280	Rate of Ki67 decreased >50% from pretreatment prostate biopsy compared with post-treatment prostatectomy tissue
Abemaciclib (2021)	II	RT + ADT	NCT04298983	Clinical response rate ^1^
Abemaciclib (2023)	I/II	Abemaciclib with darolutamide in CRPC (phase 1) and as neoadjuvant therapy in high-risk disease prior to prostatectomy (phase 2)	NCT05617885	Phase 1: MTD and RP2D Phase 2: Pathological response rate
Palbociclib (2017)	II	mCRPC after >2 lines	NCT02905318	Clinical benefit rate ^2^
Palbociclib (2015)	II	Tumor agnostic with CCND1, -2, or -3 amplification and Rb expression by IHC	NCT02465060	ORR
Abemaciclib (2024)	Ib	Abemaciclib with darolutamide in mCRPC	NCT05999968	Serious adverse event rate
Ribociclib (2015)	I/II	Ribociclib plus enzalutamide in mCRPC	NCT02555189	DLT, PSA50 ^3^
TQB3616 (2021)	Ib/II	TQB3616 with abiraterone in mCRPC	NCT05156450	12-month rPFS
Darxicilib (2024)	II	Darxicilib with abiraterone in mCRPC	NCT06500247	12-week PSA50 ^3^
PF-07220060 (2020)	I/IIa	In combination with enzalutamide mCRPC	NCT04557449	DLT, AEs, DDI, food effect
Abemaciclib (2021)	II	Abemaciclib with or without atezolizumab in mCRPC	NCT04751929	PFS, ORR, DLT, AEs
Abemaciclib (2022)	I/II	mCRPC prior to Lu-PSMA	NCT05113537	RP2D, DLT, change in SUVmax on Ga PSMA PET
Palbociclib (2020)	Ib	Talazoparib and palbociclib in solid tumors with HRR mutations such as BRCA1/2	NCT04693468	AEs and DLT

Abbreviations: AEs, adverse events; DLT, dose-limiting toxicity; DDI, Drug–drug interaction; MTD, maximum tolerated dose; RP2D, recommended phase 2 dose; mCSPC, metastatic castration-sensitive prostate cancer; mCRPC, metastatic castration-resistant prostate cancer; ORR, objective response rate; PFS, progression-free survival; rPFS, radiographic progression-free survival; SUV, standardized uptake value. ^1^: Clinical response rate will be assessed by the percentage of patients who achieve PSA nadir levels of <0.5 ng/mL on treatment. ^2^: Clinical benefit rate defined as one of the following: PSA decline >50%, complete response, or partial response or stable disease for more than 12 weeks. ^3^: PSA50 is defined as the proportion of patients with greater than or equal to 50% reduction in PSA level.

**Table 2:** Published clinical trials of CDK4/6 inhibitors in prostate cancer.

Agent	Clinical Phase	Patient Population	ClinicalTrials.gov Identifier/Reference	Primary Outcome
Palbociclib	II	RCT of ADT with or without palbociclib in Rb-positive mCSPC	NCT02059213 [79]	PSA response rate did not differ between groups.
Abemaciclib	II	Single-arm study of single-agent abemaciclib in mCRPC	NCT04408924 [80]	ORR of 6.8%
Ribociclib	Ib/II	Single-arm study of ribociclib + docetaxel in patients with mCRPC	NCT02494921 [81]	6-month rPFS was 68.5%, median rPFS 8.1 mo
Abemaciclib	III	Abiraterone with or without abemaciclib as first-line therapy in patients with mCRPC	NCT03706365 [82]	rPFS did not differ significantly between groups

Abbreviations: mCSPC, metastatic castration-sensitive prostate cancer; mCRPC, metastatic castration-resistant prostate cancer; ORR, objective response rate; rPFS, radiographic progression-free survival.

**Table 3:** Active clinical trials of PI3K/AKT, aurora kinase, and MAPK pathway inhibitors in prostate cancer.

Agent (Trial Start Date)	Clinical Phase	Target	Patient Population	ClinicalTrials.gov Identifier	Primary Outcome
Gedatolisib (2024)	I/II	PI3K/mTOR	Gedatolisib + darolutamide in mCRPC	NCT06190899	Safety, RP2D, rPFS
AZD8186 (2018)	I	PIK3b	AZD8186 + docetaxel in PTEN/PIK3CB mutated solid tumors	NCT03218826	DLT, AEs
Capivasertib (2022)	III	AKT	RCT of capivasertib + docetaxel in mCRPC	NCT05348577	OS
MK2206 (2011)	I	AKT	MK2206 with hydroxychloroquine in solid tumors, including prostate cancer	NCT01480154	MTD, DLT
MK2206 (2010)	II	AKT	MK2206 with bicalutamide in prostate cancer with evidence of biochemical recurrence	NCT01251861	Proportion of patients with undetectable PSA at 44 weeks
TAS0612 (2020)	I	AKT	Solid tumors, including mCRPC	NCT04586270	DLT, rPFS
Tinengotinib (2024)	Ib/II	Aurora A/B	Tinengotinib with abiraterone or enzalutamide in mCRPC	NCT06457919	RP2D, ORR
Trametinib	II	MEK	Single-arm study of trametinib in mCRPC	NCT02881242	PSA response rate, RECIST response rate ^1^.

Abbreviations: AEs, adverse events; DLT, dose-limiting toxicity; MTD, maximum tolerated dose; RP2D, recommended phase 2 dose; FFS, failure-free survival; mCRPC, metastatic castration-resistant prostate cancer; ORR, objective response rate; rPFS, radiographic progression-free survival; OS, overall survival. ^1^: Recist response rate defined as a decline in PSA of 30% or more, any decline in PSA of 50% or more, partial or complete response at 12 weeks, and freedom from radiographic progression at 24 weeks.

**Table 4:** Published clinical trials of CDK2, CDK7, CDK9, and PI3K/AKT inhibitors in prostate cancer.

Agent	Clinical Phase	Target	Patient Population	ClinicalTrials.gov Identifier/Reference	Primary Outcome
Flavopiridol	II	CDK1, -2, -4, and -6	Single-arm study in mCRPC	NCT00003256 [96]	No objective responses were observed; most common toxicity was nausea and diarrhea
XL-102	I	CDK7	Solid tumors, including mCRPC	NCT04726332 [97]	MTD not determined; no DLT observed
Samuraciclib	I	CDK7	Solid tumors, including CRPC	NCT03363893 [98]	360 mg was MTD, with further escalation limited by GI toxicity. Two PC patients with durable PSA reductions.
PRT2527	I	CDK9	Solid tumors, including CRPC	NCT05159518 [99]	MTD not reached; DLT not observed
Ipatasertib	II	AKT	RCT of ipatasertib + abiraterone in mCRPC	NCT01485861 [100]	rPFS improved in patients with PTEN loss
Samotolisib	Ib/II	PI3K/mTOR	RCT of Samotolisib and enzalutamide in mCRPC	NCT02407054 [101]	rPFS 10.2 vs. 5.5 mo

Abbreviations: DLT, dose-limiting toxicity; MTD, maximum tolerated dose; mCRPC, metastatic castration-resistant prostate cancer; rPFS, radiographic progression-free survival.
